# Single-cell transcriptomics identifies FOSL1-regulated IGFBP3+ melanoma subtype as a neuro-immunoregulatory signaling hub facilitating tumor progression

**DOI:** 10.3389/fimmu.2025.1662869

**Published:** 2025-11-26

**Authors:** Wenjia Ge, Ziwei Zhang, Wenjie Chen, Zhijie Zhao, Huabao Cai, Yantao Ding, Jin Xu

**Affiliations:** 1 Department of Plastic Surgery, Tongren Hospital, Shanghai Jiao Tong University School of Medicine, Shanghai, China; 2 Department of Plastic and Reconstructive Surgery, Shanghai General Hospital, Shanghai Jiao Tong University School of Medicine, Shanghai, China; 3 Department of Plastic Surgery, The First Affiliated Hospital of Anhui Medical University, Hefei, Anhui, China; 4 Department of Plastic and Reconstructive Surgery, Shanghai Ninth People’s Hospital, School of Medicine, Shanghai JiaoTong University, Shanghai, China; 5 Department of Neurosurgery, The First Affiliated Hospital of Anhui Medical University, Hefei, Anhui, China; 6 Center for Scientific Research of Anhui Medical University, Anhui Medical University, Hefei, Anhui, China; 7 Institute of Health and Medicine, Hefei Comprehensive National Science Center, Hefei, Anhui, China; 8 Department of Dermatology, The First Affiliated Hospital, Anhui, Medical University, Hefei, Anhui, China; 9 Key Laboratory of Dermatology (Anhui Medical University), Ministry of Education, Hefei, Anhui, China

**Keywords:** melanoma, single-cell sequencing, tumor microenvironment, neuro-immunity, novel biomarker

## Abstract

**Background:**

The most aggressive type of skin cancer, melanoma, has a high prevalence of metastases and a poor prognosis. Despite advancements in treatment, drug resistance and tumor microenvironment heterogeneity, especially involving neuro-immune interactions, continue to exist. The goal of this study is to uncover the cellular heterogeneity of melanoma in order to pinpoint molecular targets and tumor-promoting subtypes.

**Methods:**

Melanoma single-cell RNA sequencing data came from GEO. Twelve cell types were discovered after Harmony batch effect adjustment and Seurat quality control; melanoma cells were subtyped. Functional expression of differential genes was examined using gene ontology and gene set enrichment. Cytotrace measured subtype differentiation potency. PySCENIC revealed transcription factor regulatory networks, and CellChat predicted intercellular communication between malignant cell subtypes and other cell types. Functional experiments with A375 and MEWo cell lines—lentiviral shRNA knockdown, CCK-8 proliferation, wound healing, transwell migration, and flow cytometry apoptotic assays—confirmed the analysis.

**Results:**

Single-cell RNA sequencing was utilized to analyze melanoma cell subtypes and their interactions in the tumor microenvironment. C2 *IGFBP3*+, which had the lowest CytoTRACE2 score and was enriched in late tumor stages, affected melanoma development. This subtype expressed prominent immunomodulatory pathways. The C2 subtype mediate signaling to fibroblasts/pericytes via the PROS pathway and myeloid/plasmacytoid dendritic cells via the MHC-II pathway. The C2 subtype was strongly linked with FOSL1 expression, which may affect gene transcription and illness progression. FOSL1 knockdown significantly increased apoptosis and decreased melanoma cell motility and proliferation *in vitro*.

**Conclusion:**

We identified immunoregulatory C2 *IGFBP3*+ melanoma cell subtypes in our investigation, and FOSL1 was a crucial transcription factor that aided in cell migration, proliferation, and survival. Because the C2 subtype involves the MHC-II and PROS signaling pathways, which have been shown to have roles in neuroimmunology, neuroinflammation, and pain regulation, it may serve as a hub for neuro-immune interactions in the tumor microenvironment. Precision treatments for melanoma may be advanced by focusing on the FOSL1 or C2 subtype pathways, which may assist in overcoming immunotherapy resistance.

## 1 Introduction

Melanoma is an extremely aggressive form of skin cancer, with its frequency showing a steady increase in recent decades. The risk factors for melanoma include environmental variables like ultraviolet radiation exposure and internal factors such as family history, a history of atypical moles, and the number of moles (1). Melanoma arises from neural crest-derived melanocytes, establishing an intrinsic connection to the nervous system (2). Solid tumors, including melanoma, are increasingly recognized to be innervated, which plays complex roles in tumor progression, metastasis, and modulation of the tumor microenvironment (TME), including immune responses (3–6). Bidirectional communication between nerves, immune cells, and cancer cells forms the emerging field of cancer neuroimmunology.

The clinical manifestation of melanoma is significantly diverse, whereas original tumors typically appear on the skin, melanoma may also develop in ocular areas and mucous membranes or show as metastatic illness without a discernible underlying cutaneous lesion (7). The prognosis for melanoma is predominantly poor, especially in advanced stages, as people frequently pursue medical advice at these later phases (8). Surgical surgery is the fundamental treatment for early-stage melanoma (1). Nevertheless, atypical presentations such as amelanotic melanoma, provide diagnostic difficulties due to their possibility of clinical misdiagnosis, while maintaining a mortality risk similar to that of pigmented melanoma (9). Furthermore, melanomas detected via self-examination are generally thicker, more prone to ulceration, exhibit a greater likelihood of spreading and associated with a heightened rate of melanoma-related mortality (8, 10).

Most conventional treatment options for melanoma include radiation therapy, chemotherapy, and surgical excision (11). However, these conventional treatments occasionally have limited success in treating metastatic melanoma, and they frequently do not significantly improve patient survival rates (12–15). Although surgical surgery is frequently recommended for early-stage melanoma, the cancer’s efficacy significantly declines once it has metastasized (12, 16). Chemotherapeutic drugs, like dacarbazine and paclitaxel, demonstrate minimal efficacy in melanoma treatment and are often linked to significant side effects (17, 18).

Recent treatment approaches have been developed in response to recent discoveries in discovering the molecular basis of melanoma (19, 20). In the treatment of melanoma, targeted therapy and immunotherapy have come to be necessary because they obstruct changes in genes like BRAF and MEK, preventing the growth and spread of tumor cells (21–23). However, targeted therapy’s efficacy is frequently only temporary because many patients develop drug resistance shortly after starting treatment, leading to tumor recurrence (17, 24, 25). The therapy’s efficacy is limited in individuals without BRAF mutations, leading to a group of patients who do not benefit from the treatment (26, 27). Therefore, the administration of various targeted therapies in conjunction is crucial to delay the emergence of resistance (24, 28).

Immunotherapy operates by stimulating the patient’s immune system to identify and attack tumor cells (29–31). Recent melanoma immunotherapy progress is significant, especially with the use of immune checkpoint inhibitors (ICIs), including anti- PD- 1 agents (e. g., Nivolumab and Pembrolizumab) and anti- CTLA- 4 agents (e. g., Ipilimumab), which have shown a marked improvement in survival rates for melanoma patients (32, 33). By reactivating T cells, these medications increase the immune system’s ability to recognize and treat malignancies (21, 22). A significant number of patients continue to receive immunotherapy despite the ongoing advantages that some patients have (34, 35). A significant obstacle in melanoma immunotherapy is the heterogeneity and intricacy of the tumor microenvironment, where the interactions between immune cells, stromal cells and tumor cells in the tumor microenvironment significantly affect the effectiveness of therapeutic interventions (36). Conventional biomarkers for immunotherapy, such as tumor mutation burden and PD-L1 expression, have shown poor reliability when predicting patient responses to treatment (37). To address these issues, researchers are looking into the use of precise care and immunotherapy (38, 39). Although its reply rate is relatively lower, immunotherapy possesses the potential for enduring lesion management. Recent research indicates that the integration of targeted therapy with immunotherapy may augment the therapeutic effectiveness of immunotherapy, potentially yielding a synergistic anti-tumor impact (40–43). The nervous system is a significant but little understood TME component besides the well-studied interactions between tumor, immune, and stromal cells (44, 45). Neural activity, neurotransmitters, neurotrophic factors and neuroinflammation could profoundly influence tumor immunity, potentially contributing to immunotherapy resistance and disease progression (46). Understanding the interplay between tumor cell heterogeneity, particularly immunomodulatory subtypes and neural components within the TME is crucial for developing more effective therapeutic strategies (47).

Single-cell RNA sequencing (scRNA-seq) technology offers new perspectives on tumor heterogeneity and microenvironment dynamics. In order to improve our understanding of tumor growth, immune responses, and treatment resistance, the sophisticated analytical method enables the identification of rare cell populations, co-expression patterns, and regulatory networks (48–51). Single-cell analysis has been used to study the immune system’s environment in melanoma, enabling the identification of novel therapeutic targets and the prediction of biomarkers (52, 53). The incorporation of bioinformatics analysis enables single-cell technologies to find genetic indicators linked to prognosis, facilitate the development of novel therapeutic targets and eventually inform clinical treatment decisions (54–58).

Motivated by these discoveries and the increasing importance of the neuro-immune axis in cancer, we performed a scRNA-seq analysis on melanoma cells to further clarify the tumor’s cellular heterogeneity and microenvironment. By mapping the transcriptomes of distinct cell populations, we aim not only to identify immunomodulatory tumor subtypes and key regulators but also to explore potential intersections between these findings and neural influences within the TME, providing a more holistic view of melanoma microenvironment complexity. This work intends to create a basis for customized strategies to tackle the therapeutic difficulties presented by melanoma.

## 2 Materials and methods

### 2.1 Single-cell data acquisition

The dataset for single-cell melanoma analysis was obtained from the GEO database (https://www.ncbi.nlm.nih.gov/geo/) with accession number GSE277165. The dataset for study comprised melanoma samples from ten biopsies. Ethical approval was unnecessary since the study utilized publicly accessible database information.

### 2.2 Data processing and visualization

R (v 4.3.3) and the Seurat analysis framework (v 4.4.0) were employed to examine the raw data (59, 60). We first detected and removed doublet cells utilizing the DoubletFinder algorithm (v 2.0.3) (61). To select high-quality single cells, we implemented rigorous quality control measures: the total UMI count (nCount) ranged from 500 to 100,000, the number of detected genes per cell (nFeature) was limited to 300-7500, mitochondrial gene expression was restricted to below 25%, and erythrocyte gene expression was confined to under 5%. The “NormalizeData” function was employed to standardize the data, and the top 2,000 highly variable genes were identified from the normalized expression matrix utilizing “FindVariableFeatures” (62, 63). The expression values were normalized utilizing the “ScaleData” function (64, 65). Subsequent to principal component analysis (PCA), batch effects were corrected using the Harmony package (v 0.1.1) (66, 67), and the first 30 principal components were selected for Uniform Manifold Approximation and Projection (UMAP) dimensionality reduction and visualization, yielding spatial distribution maps (68). For cell type identification, we referenced the CellMarker database and assigned appropriate markers to the detected cell clusters, determining the distribution and fraction of various cell types (69, 70). To examine the heterogeneity of melanoma, we reclustered the melanoma cells and annotated each subgroup based on their unique marker genes.

### 2.3 Cellular preference and functional enrichment analysis

The Odds Ratio (OR) model was employed to evaluate the stage specificity of cells in different biopsy samples across several primary melanoma stages (Group I: stage I, Group II: stage II, and Group III: stage III) (71). We subsequently utilized the Wilcoxon rank-sum test using the default parameters of the “FindAllMarkers” algorithm (Log FC > 0.25) to identify differentially expressed genes (DEGs) for each cell population and subpopulation. The ClusterProfiler (v4.6.2) and SCP (v0.4.8) packages were employed for Gene Ontology Biological Process (GOBP) analysis, alongside Gene Set Enrichment Analysis (GSEA) to examine the co-expression patterns of gene modules (72, 73). To investigate dynamic functional activity at the single-cell level, the AUCell method was utilized to evaluate the enrichment of stem cell-related gene sets, rating cells through the “AUCell_buildRankings” function (74).

### 2.4 Cellular differentiation analysis

To investigate the differences in developmental and differentiation state among melanoma subpopulations, we assessed cell stemness utilizing the CytoTRACE2 R package (v 0.3.3) (75). A KNN graph illustrating the undirected interactions among cells was initially generated. CytoTRACE2 was later utilized to determine the ideal temporal sequence of cells. The k-nearest neighbors (KNN) graph and specified time were subsequently utilized to construct a transfer matrix, which was later represented on a UMAP scatter plot.

### 2.5 Intercellular communication network establishment

We performed a thorough investigation of the cellular contact network in the disease microenvironment utilizing the CellChat software (v 1.6.1) (76). By integrating ligand-receptor interaction datasets, we predicted essential signaling pathway functions. The “netVisual_diffInteraction” function was used to demonstrate the differences in communication strength among subtypes, while the “identifyCommunicationPatterns” function was employed to determine the number of communication patterns. The CellChatDB database (http://www.cellchat.org/) was employed to identify relevant signaling pathways and receptor interactions. A p-value threshold of 0.05 was set to determine biologically significant intercellular communication events.

### 2.6 Gene regulatory network by SCENIC

Utilizing the SCENIC software package (v0.10.0) in a Python 3.7 environment, we reconstructed the gene regulatory network from scRNA-seq data and found stable cellular states. The gene regulatory network was constructed using co-expression and DNA motif analysis. The cell state was ascertained by examining the network’s activity in each cell. We methodically evaluated the enrichment and regulatory function of transcription factors (TFs) by creating an AUCell matrix (77).

### 2.7 Cell culture

The human melanoma cell lines A375 and MEWo were acquired from the Cell Bank of the Chinese Academy of Sciences in Shanghai, China, and were authenticated using STR profiling. Cells were grown in RPMI-1640 media (Thermo Fisher Scientific, USA) augmented with 10% fetal bovine serum (FBS, Thermo Fisher Scientific, USA) and 1% penicillin-streptomycin (Thermo Fisher Scientific, USA) at 37 °C in a humidified environment with 5% CO_2_.

### 2.8 Lentiviral transduction and FOSL1 knockdown

Lentiviral vectors encoding shRNAs (GenePharma, Shanghai, China) targeting FOSL1 (sh-FOSL1–1 and sh-FOSL1-2) and control vector (shCtrl) were transduced into A375 and MEWo cells utilizing Lipofectamine 3000 (Thermo Fisher Scientific, USA), in accordance with the manufacturer’s guidelines. Following a 48-hour period, cells were subjected to selection using puromycin (1 μg/mL, Thermo Fisher Scientific, USA) for a duration of 5 days. The knockdown efficiency was confirmed using qRT-PCR and Western blot analysis.

### 2.9 Quantitative real-time PCR

Total RNA was obtained from A375 and MEWo cells utilizing TRIzol™ reagent (Thermo Fisher Scientific, USA) in accordance with the manufacturer’s guidelines. The purity and concentration of RNA were evaluated using a NanoDrop 2000 spectrophotometer (Thermo Fisher Scientific, USA). For cDNA synthesis, 1 μg of total RNA was reverse transcribed utilizing the PrimeScript™ RT Reagent Kit (Takara, Japan) with gDNA Eraser to eliminate genomic DNA contamination.

Quantitative reverse transcription polymerase chain reaction (qRT-PCR) was conducted utilizing TB Green™ Premix Ex Taq™ II (RR820A, Takara, Japan) on a QuantStudio 5 Real-Time PCR System (Applied Biosystems, USA). The thermal cycling parameters were as follows: 95°C for 30 seconds, succeeded by 40 cycles of 95°C for 5 seconds and 60°C for 30 seconds. A melting curve study was conducted to verify amplification specificity.

The relative expression of FOSL1 was determined utilizing the 2^–ΔΔCt technique, employing GAPDH as the internal control. All reactions were conducted in triplicate. The primer sequences are specified in **Supplementary Table 1**.

### 2.10 Western blotting

Upon achieving around 70% confluence post-transfection, the cells were lysed using RIPA buffer for collection. The resultant lysates underwent centrifuged at 12,000 rpm for 15 minutes to eliminate cellular debris, and the supernatants were harvested for further protein analysis via SDS-PAGE. The isolated proteins were subsequently transferred to PVDF membranes and treated with 5% bovine serum albumin (BSA) for 1.5 hours at ambient temperature to reduce non-specific interactions. Subsequently, the membranes were treated with the primary antibody (Cell Signaling Technology, Cat# 5281 for FOSL1; Cat# 5174 for GAPDH) overnight at 4 °C, followed by a one-hour incubation with a horseradish peroxidase-conjugated secondary antibody. Protein detection was performed using an improved chemiluminescence substrate for Western blotting.

### 2.11 Cell proliferation assay (CCK-8)

Transduced A375 and MEWo cells were plated in quintuplicate at a density of 2×10³ cells per well in 96-well plates. Cell viability was assessed from days 1 to 5 with the CCK-8 kit (Dojindo Laboratories, Kumamoto, Japan). At each time interval, 10 µL of CCK-8 reagent was introduced to each well and incubated for 2 hours. Absorbance at 450 nm was quantified utilizing a microplate reader (BioTek Instruments).

### 2.12 Colony formation assay

Cells (500 per well) were inoculated in 6-well plates and cultivated for 10 to 14 days. Colonies were treated with 4% paraformaldehyde (Beyotime Biotechnology, Shanghai, China) for 15 minutes, stained with crystal violet (Beyotime Biotechnology, Shanghai, China) for 30 minutes, and subsequently photographed. Colonies including above 50 cells were counted manually.

### 2.13 Wound healing assay

Cells were inoculated into 6-well plates and cultivated to complete confluence. A sterile 200 µL pipette tip was employed to generate a linear incision across the cell monolayer. Following PBS washing to eliminate debris, cells were cultured in serum-free media. Wound closure was documented at 0 hours and 72 hours using an inverted microscope (Leica DMi8), and wound width was quantified with ImageJ software.

### 2.14 Transwell migration assay

Cell migration was evaluated utilizing transwell chambers (8 µm hole size, Corning, USA). 5×10^4^ cells in 200 µL of serum-free media were introduced into the upper chamber, while 600 µL of medium containing 10% FBS was allocated to the lower chamber. Following 24 hours of incubation, migrating cells on the lower membrane surface were fixed using 4% paraformaldehyde, stained with 0.5% crystal violet, and enumerated in five random fields under a microscope.

### 2.15 Apoptosis assay (Flow cytometry)

Apoptosis was evaluated with Annexin V-FITC/PI double labeling (BD Biosciences, San Jose, USA). Cells were collected, rinsed with cold PBS, and resuspended in 1× binding buffer. Annexin V-FITC and PI were administered following the manufacturer’s procedure, and samples were incubated in the dark for 15 minutes. Stained cells were examined with a BD FACSCanto II flow cytometer, and the data were processed using FlowJo software (v10.8).

### 2.16 Statistical analysis

Statistical analysis was performed using R software (v4.3.0) and Python software (v4.2.0). The Wilcoxon test and Pearson correlation coefficient were utilized to assess the significance of differences between groups. Results were considered statistically significant when *P* < 0.05.

## 3 Results

### 3.1 Development of the single-cell transcriptomic atlas for melanoma

**Figure 1:**
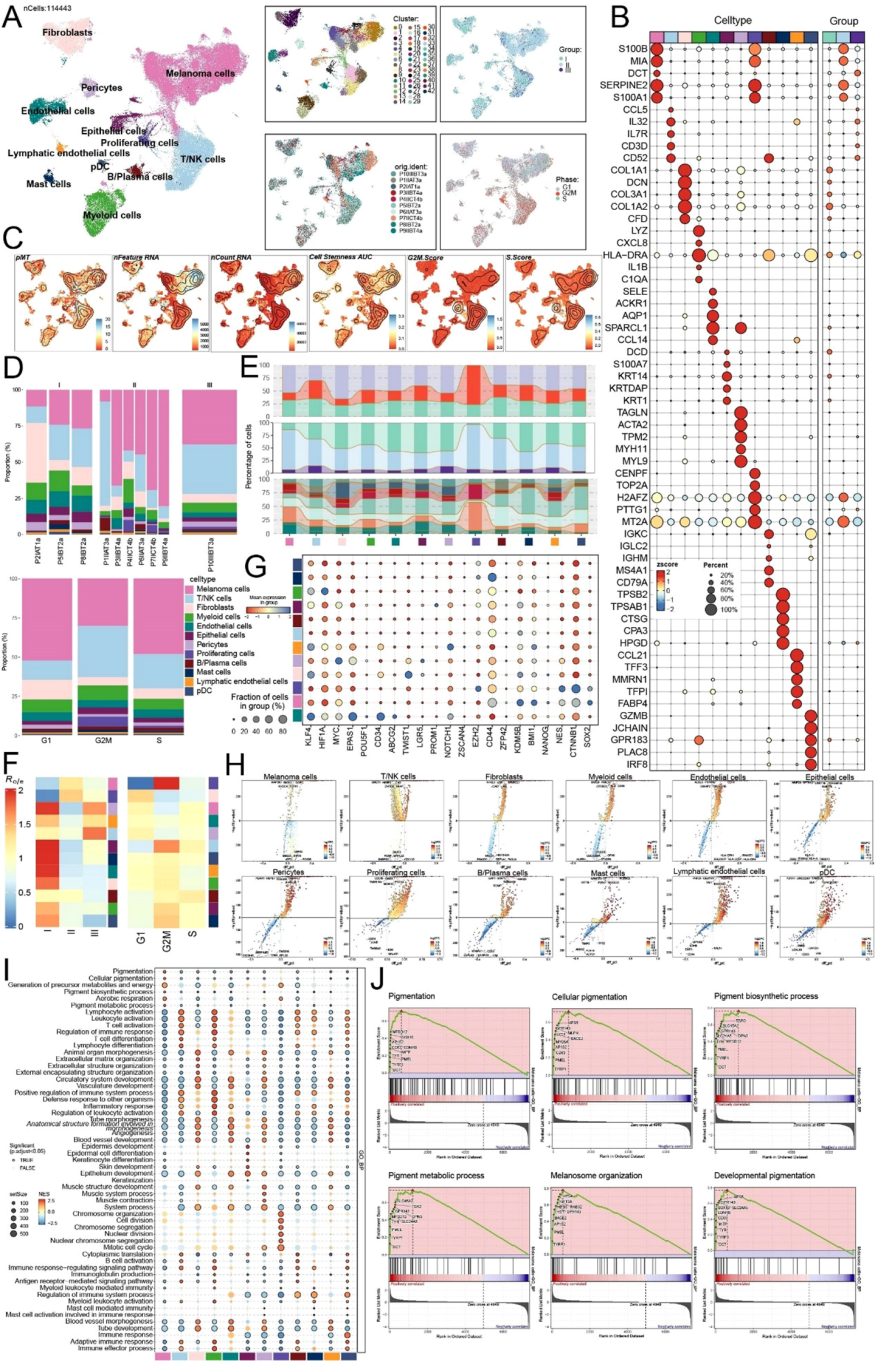
Exploring melanoma heterogeneity through scRNA-seq analysis. **(A)** UMAP visualization of the distribution characteristics of all cell samples based on specific marker genes for cell type identification and annotation. The four-panel figure on the right showed the overall distributions of the clusters, sample originals, tissue groups (Group I, Group II and Group III), and cell phases (G1, G2/M and S phase). **(B)** Bubble plot analyzed the average expression levels of the top five marker genes in each cell type and group, with bubble size proportional to gene expression percentage, and the color gradient indicating data normalization. **(C)** Distribution characteristics of pMT, nFeature RNA, nCount RNA, Cell Stemness AUC, G2/M Score, and S Score. **(D)** Stacked bar charts quantified the differences in cell composition among the groups (upper) and phases (lower). **(E)** Quantifications of the phases (upper), groups (middle) and originals (lower) in each cell type **(F)** Ro/e scores revealed the preference of cell types for different groups (left) and phases (right). **(G)** Bubble plot revealed the differential expression of stemness feature genes across different cell types. **(H)** Volcano plot visualized DEGs in various melanoma cell types, highlighting the top five upregulated and downregulated genes in each cell type (P-adj < 0.05), with dashed lines marking the significance boundary. **(I)** Bubble plot using GSEA enrichment analysis with GOBP terms of different biological processes for each cell type.**(J)** GSEA enrichment analysis of melanoma cells.

This research created a single-cell transcriptome map of melanoma using data from the GEO database (GSE277165), which includes scRNA-seq data from ten samples. After stringent quality control and unsupervised clustering, 114,443 high-quality cells were retained to characterize the cellular topography of initial melanoma. **Figure 1A** depicted the clustering results of all cells. By employing canonical marker genes and their unique expression profiles, we accurately identified 12 distinct cell types: melanoma cells, T/NK cells, fibroblasts, myeloid cells, endothelial cells, epithelial cells, pericytes, proliferating cells, B/plasma cells, mast cells, lymphatic endothelial cells, and plasmacytoid dendritic cells (pDCs). Samples from stage I, II, and III melanoma were categorized into Groups I, II, and III, respectively. Melanoma cells and T/NK cells were significantly abundant in the stage II and III cohorts. Furthermore, cell cycle analysis revealed that melanoma cell subtypes exhibited the highest proportion of cells in the G1 and S phases. Differential gene expression analysis among groups identified a set of melanoma cell-specific marker genes (*S100B*, *MIA*, *DCT*, *SERPINE2*, and *S100A1*) (**Figure 1B**), most of which were considerably upregulated in Group II. We subsequently conducted an examination of the distribution and densities of 12 cell types expressing pMT, nFeature RNA, nCount RNA, cell stemness AUC, G2/M score, and S score (**Figure 1C**). Cell proportion analysis confirmed that melanoma cells and T/NK cells were the principal constituents of Groups II and III, with a majority of melanoma cells in the G1 phase (**Figure 1D**). By analyzing phase distributions, groups, and originals across 12 cell types, we established that most melanoma cells were located in the G1 phase, Group II, and derived from T4b tumor stage samples (**Figure 1E**). Furthermore, the Ro/e (observed/expected ratio) preference study independently validated the enrichment of melanoma cells in Group II and further suggested that melanoma cells exhibited a greater propensity to occupy the G1 and S phases (**Figure 1F**).

### 3.2 Single-cell sequencing disclosed melanoma cellular populations’ differential stemness and functional enrichment profiles

Stemness is characterized by the ability to maintain an undifferentiated state, perform self-renewal, and differentiate into several lineages. To investigate the differences in stem cell attributes among classified cell types in melanoma, we utilized a bubble plot to visually depict the expression profiles of stemness-related genes across various cell types. The results revealed that melanoma cells exhibited substantial expression of critical stemness-associated genes, namely *HIF1A, EPAS1, TWIST1* and *EZH2* (**Figure 1G**). Furthermore, we utilized volcano plots to highlight the five most significantly upregulated and downregulated genes among the twelve identified cell types (**Figure 1H**). In melanoma cells, the elevated genes included *MAP2K7, RIOK1, GOPC, KHDC4* and *PDPK1*, which may be associated with stress/drug resistance, protein synthesis, and the regulation of cellular migration. Conversely, the downregulated genes, including as *ATF2, MED21, USP33, STAT6* and *PDS5B*, may signify repression or functional deficiencies in DNA repair, differentiation, and microenvironment remodeling. We performed a series of thorough enrichment studies based on differentially expressed genes across cell types. The GSEA results demonstrated a notable enrichment of melanoma cells in pathways related to melanin synthesis and deposition, specifically “Pigmentation,” “Cellular pigmentation,” “Pigment biosynthetic process,” “Pigment metabolic process,” “Melanosome organization,” and “Developmental pigmentation” (**Figures 1I, J**). These findings provided molecular evidence that highlights the disruption of melanocytic differentiation and genomic stability in the melanoma tumor microenvironment, offering crucial insights and a mechanistic foundation for future research on melanoma progression and therapeutic resistance.

### 3.3 Identifying and characterizing subtypes of melanoma cells

**Figure 2:**
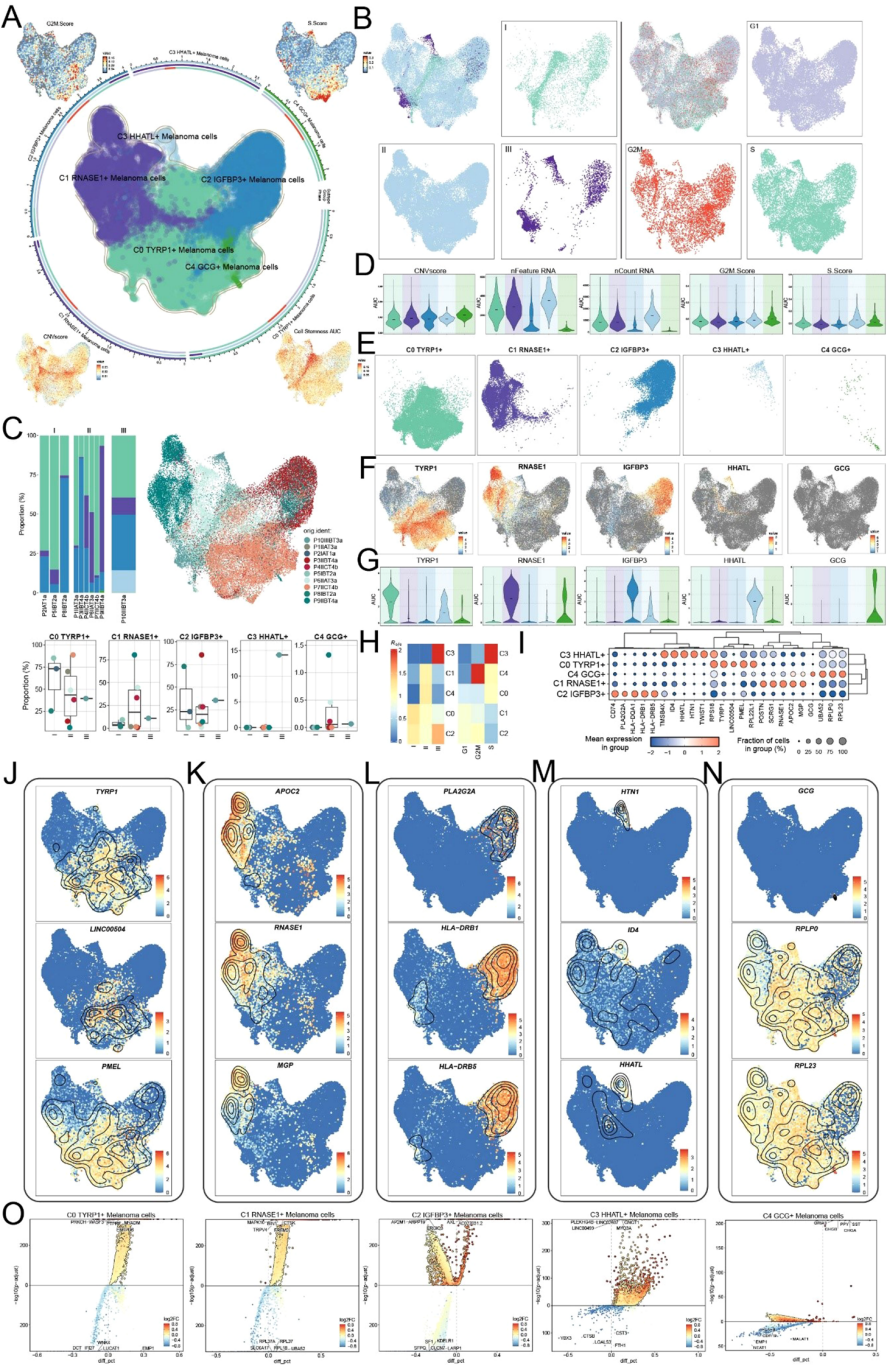
Characterization of melanoma cell subtypes profiles. **(A)** UMAP plots presented the clustering distribution characteristics of five distinct melanoma cell subtypes identified based on differential marker gene expression, with contour lines outlining the boundaries of each subtype. The outer axis represented the log scale of each neutrophil subpopulation. The three-ring annotation layer encoded subtype (outer ring), groups (middle ring), and cell phase stage (inner ring). UMAP plots, arranged in the four corners and proceeding clockwise from the upper left corner, illustrated the expression distribution of G2M scores, S scores, Cell Stemness AUC, CNV scores across all melanoma cells. **(B)** Faceted UMAP plots compared the distribution characteristics of each group (left) and phase (right). **(C)** Distribution of the melanoma cell types from each sample original (upper left) and distributions of the originals in each melanoma cell type (upper right and lower). **(D)** Violin plots showed the AUC of CNV score, nFeature RNA, nCount RNA, G2/M Score and S Score. **(E)** Faceted UMAP plots compared the distribution characteristics of individual melanoma cell subtypes. **(F, G)** UMAP visualization of the distribution patterns of specific marker genes for each melanoma cell subtype, with violin plots showed their AUC across subtypes. **(H)** Ro/e scores revealed the preference of melanoma cell subtypes for different groups (left) and phases (right). **(I)** Bubble plot analyzed the average AUC of the top five named genes in each melanoma cell subtype, with bubble size proportional to gene expression percentage, and the color gradient indicating data normalization. **(J-N)** UMAP plots displayed the distribution patterns of the top three marker genes in all melanoma cell subtypes, with the order of left to right showing C0, C1, C2, C3 and C4. **(O)** Volcano plots visualized DEGs in melanoma cell subtypes, highlighting the top five upregulated and downregulated genes in each subtype (P-adj < 0.05), with dashed lines marking the significance boundary.

Considering the pivotal role of tumor cells in melanoma, we reexamined 47,047 melanoma cells and discerned five distinct subtypes (**Figure 2A**): the C0 subtype, characterized by elevated *TYRP1* expression; the C1 subtype, defined by heightened *RNASE1* expression; the C2 subtype, distinguished by increased *IGFBP3* expression; the C3 subtype, identified by *HHATL* expression; and the C4 subtype, recognized by *GCG* expression. UMAP faceting was utilized to methodically analyze the distribution patterns among different groups and stages (**Figure 2B**). Proportional analysis revealed an elevation of C0 *TYRP1*+ in Group I, C1 *RNASE1*+ and C4 *GCG*+ in Group II, and C2 *IGFBP3*+ and C3 *HHATL*+ in Group III (**Figure 2C**). The figure additionally depicts the distributions and proportions of subtypes originating from the original samples. We next displayed the results of CNVscore, nFeature RNA, nCount RNA, G2/M score, and S score for different tumor cell subtypes utilizing violin plots (**Figure 2D**). UMAP facing was used to methodically examine the distribution patterns of various melanoma cell subtypes, highlighting distinctive spatial characteristics for each subtype (**Figure 2E**). **Figures 2F** and **Figure 2G** illustrate the expression patterns of marker genes in melanoma cells. Ro/e preference analysis validated the enrichment of subtypes across groups and phases, indicating a higher cellular abundance of C2 and C3 subtypes in Group III, and an increased abundance of C3 and C4 in the G2M/S phase (**Figure 2H**).

To enhance the visualization of gene expression profiles, we presented the five principal marker genes for each subtype in a bubble plot (**Figure 2I**), with the top three depicted in UMAP plots (**Figures 2J-N**). The examination of volcano plots revealed significant variations in gene expression across the five subtypes (**Figure 2O**).

### 3.4 Differentiational profiles of melanoma cells revealed by stemness analysis

**Figure 3:**
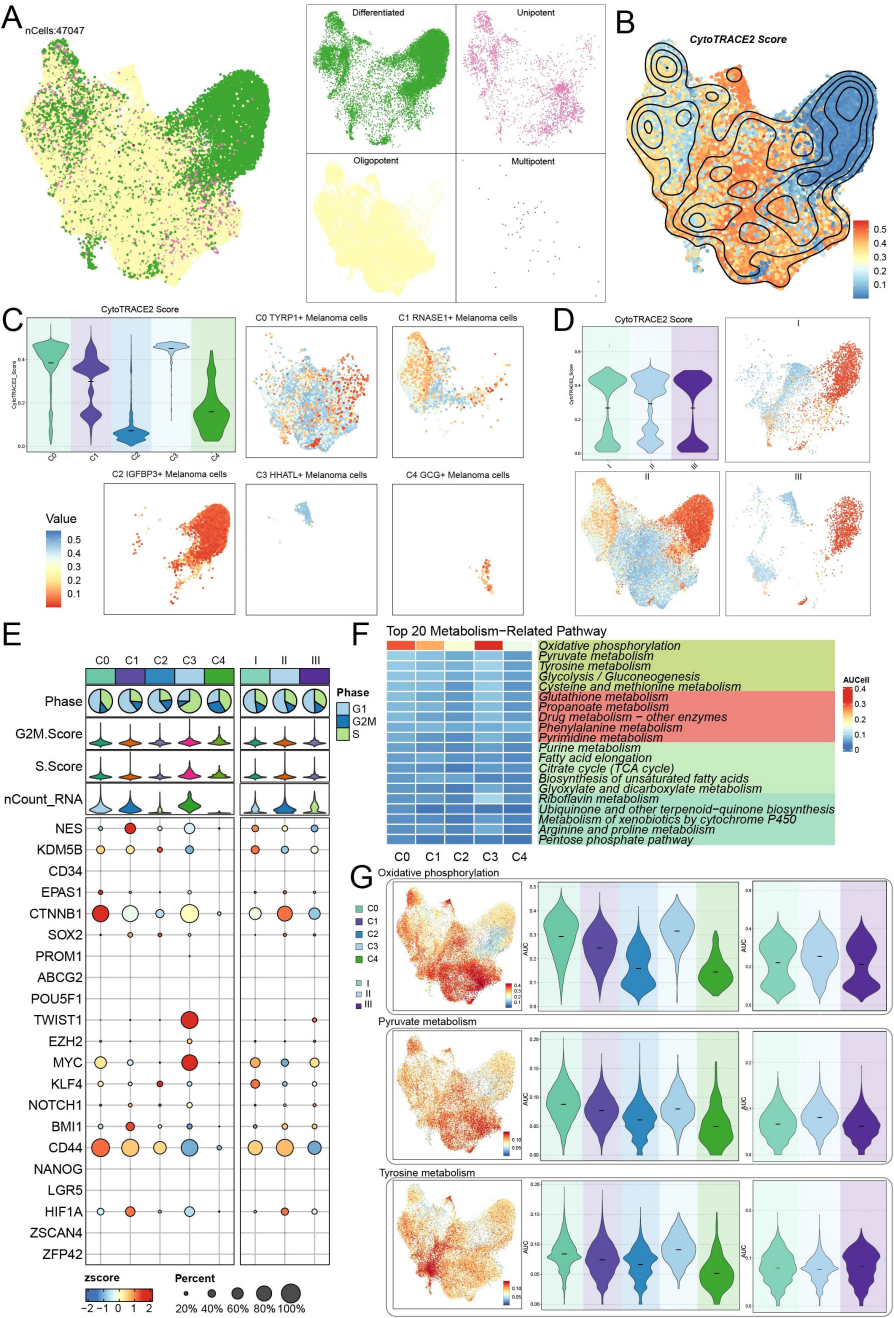
Differentiation potency and metabolic reprogramming of melanoma cells. **(A)** UMAP analysis depicted the distributions of melanoma cells with different differentiation potencies. Four panels on the right showed the individual subpopulation separately. **(B)** Distribution of CytoTRACE2 score in the melanoma cells. **(C)** The violin plot quantified CytoTRACE2 score among melanoma cell subtypes, and the faceted UMAP plots showed the distribution characteristics of individual subtypes. **(D)** The violin plot quantified CytoTRACE2 score among groups, and the faceted UMAP plots showed the distribution characteristics of individual groups. **(E)** The bubble plot illustrated the differential expression of stemness genes across various melanoma cell subtypes and groups. **(F)** Heatmap showed the top 20 metabolic pathways in the five melanoma cell subtypes. **(G)** UMAP analysis revealed the distribution patterns of significantly highly expressed metabolic pathways (oxidative phosphorylation, pyruvate metabolism and tyrosine metabolism). Violin plots further analyzed the AUCell heterogeneity of these pathways across different melanoma cell subtypes and groups.

**Figure 3A** shows a variance in the differentiation potential between melanoma cells from the CytoTRACE2. UMAP and violin plots were used to represent the CytoTRACE2 ratings for various subtypes and groups (**Figures 3B-D**). The results revealed that the C2 *IGFBP3*+ subtype was the most differentiated, as seen by a markedly lower CytoTRACE2 score, while the C3 *HHATL*+ subtype displayed the contrary tendency. No significant differences were identified across the groups. We analyzed the differential expression levels of stemness-related genes among different subtypes and groups, revealing that *KLF4* was markedly elevated in the C2 *IGFBP3*+ melanoma cells. *TWIST1* and *MYC* demonstrated substantial expression in C3 *HHATL*+ melanoma cells (**Figure 3E**).

### 3.5 Metabolic reprogramming in melanoma cells

To examine the metabolic function of melanoma cells during disease progression, we performed pathway enrichment analysis for each subtype, revealing the top 20 metabolism-related pathways (**Figure 3F**). Pathways such as oxidative phosphorylation, pyruvate metabolism, tyrosine metabolism, glycolysis/gluconeogenesis, and cysteine and methionine metabolism demonstrated particularly significant AUC scores. Additionally, UMAP plots were utilized to depict the expression distribution of oxidative phosphorylation, pyruvate metabolism, and tyrosine metabolism pathways in melanoma cells. UMAP plots and violin plots were utilized to examine the AUC ratings of these routes among different subtypes and groups (**Figure 3G**).

### 3.6 Functional enrichment in melanoma cells

**Figure 4:**
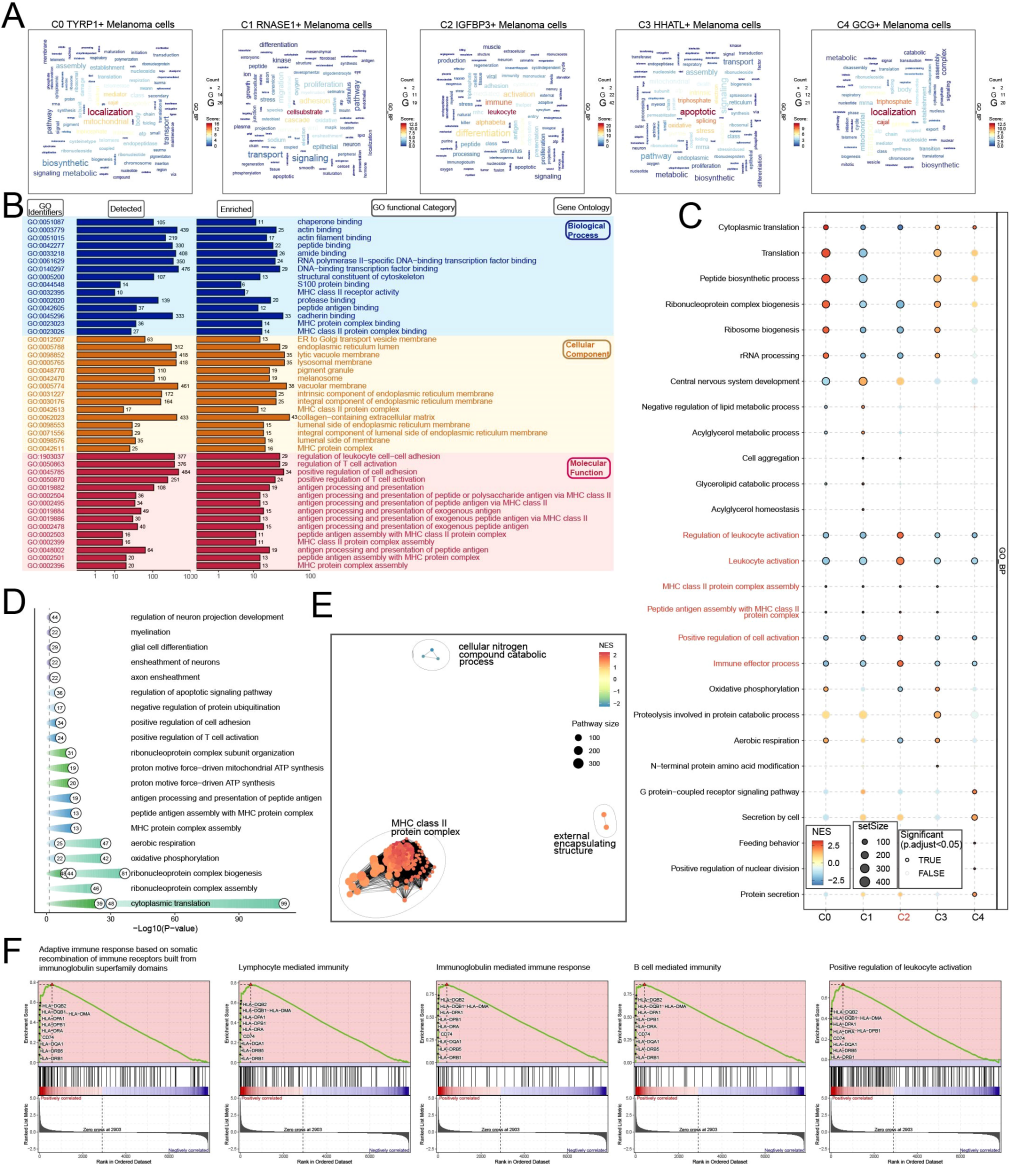
Biological features of melanoma cell subtypes based on GO/GSEA enrichment strategies. **(A)** Word cloud constructed from GOBP enrichment analysis, visually showing the enrichment degree of pathways associated with melanoma cell subtypes.**(B)** GOBP, GOCC, GOMF enrichment analysis of C2 *IGFBP3+* melanoma cell subtype.**(C)** Bubble plot showed the results of GSEA of gene sets in different melanoma cell subtypes, revealing key biological functions in each subtype.**(D)** GO analysis based on differential genes of the five melanoma cell subtypes.**(E)** Enrichment analysis in network of C2 *IGFBP3+* melanoma cell subtype revealed its key biological functions.**(F)** GSEA of C2 *IGFBP3+* melanoma cell subtype. .

After understanding the differentiation potential and metabolic pathways of melanoma cell subtypes, we performed a comprehensive investigation of their roles. Initially, GOBP analysis was conducted on each subtype. The results demonstrated that the C0 subtype was primarily linked to localization processes, the C1 subtype to cell-substrate interactions, the C2 subtype to leukocyte and immune functions, the C3 subtype to apoptotic, triphosphate, and splicing activities, and the C4 subtype to localization and Cajal processes (**Figure 4A**). Due to the C2 subtype’s distinct correlation with immunological mechanisms, we focused our research on this subtype. We performed additional investigations to elucidate the function of the C2 subtype in melanoma. We performed a comprehensive Gene Ontology analysis, concentrating on Biological Process (BP), Cellular Component (CC), and Molecular Function (MF) categories (**Figure 4B**). The C2 subtype in the BP demonstrated considerable enrichment in categories like “Cadherin binding,” “DNA-binding transcription factor binding,” and “Amide binding,” among others. The C2 subtype in the CC shown enrichment in “Collagen-containing extracellular matrix” and “Vacuolar membrane,” among other categories. The C2 subtype in the MF shown enrichment in processes including “Positive regulation of cell adhesion,” “Regulation of leukocyte cell-cell adhesion,” and “Regulation of T cell activation,” among others. GSEA was conducted on the GOBP keywords to further corroborate the enriched functionality of C2 (**Figure 4C**). The results demonstrated that the C2 subtype displayed significant specificity in the expression of terms such as “Regulation of leukocyte activation,” “Leukocyte activation,” “MHC class II protein complex assembly,” “Peptide antigen assembly with MHC class II protein complex,” “Positive regulation of cell activation,” and “Immune effector process.” Subsequent to the identification of DEGs in melanoma cells, a GO enrichment analysis was conducted (**Figure 4D**). The network diagram demonstrated a notable enrichment of functions associated with the MHC class II protein complex in the C2 subtype (**Figure 4E**). A Gene Set Enrichment Analysis (GSEA) was performed for the C2 subtype (**Figure 4F**), indicating positive enrichment trends in “Adaptive immune response based on somatic recombination of immune receptors derived from immunoglobulin superfamily domains,” “Lymphocyte-mediated immunity,” “Immunoglobulin-mediated immune response,” “B cell-mediated immunity,” and “Positive regulation of leukocyte activation.” Focusing on the C2 subtype may yield insights into the immune milieu of melanoma.

### 3.7 Signaling function of the C2 subtype within the inflammatory microenvironment elucidated by cell-cell communication

**Figure 5:**
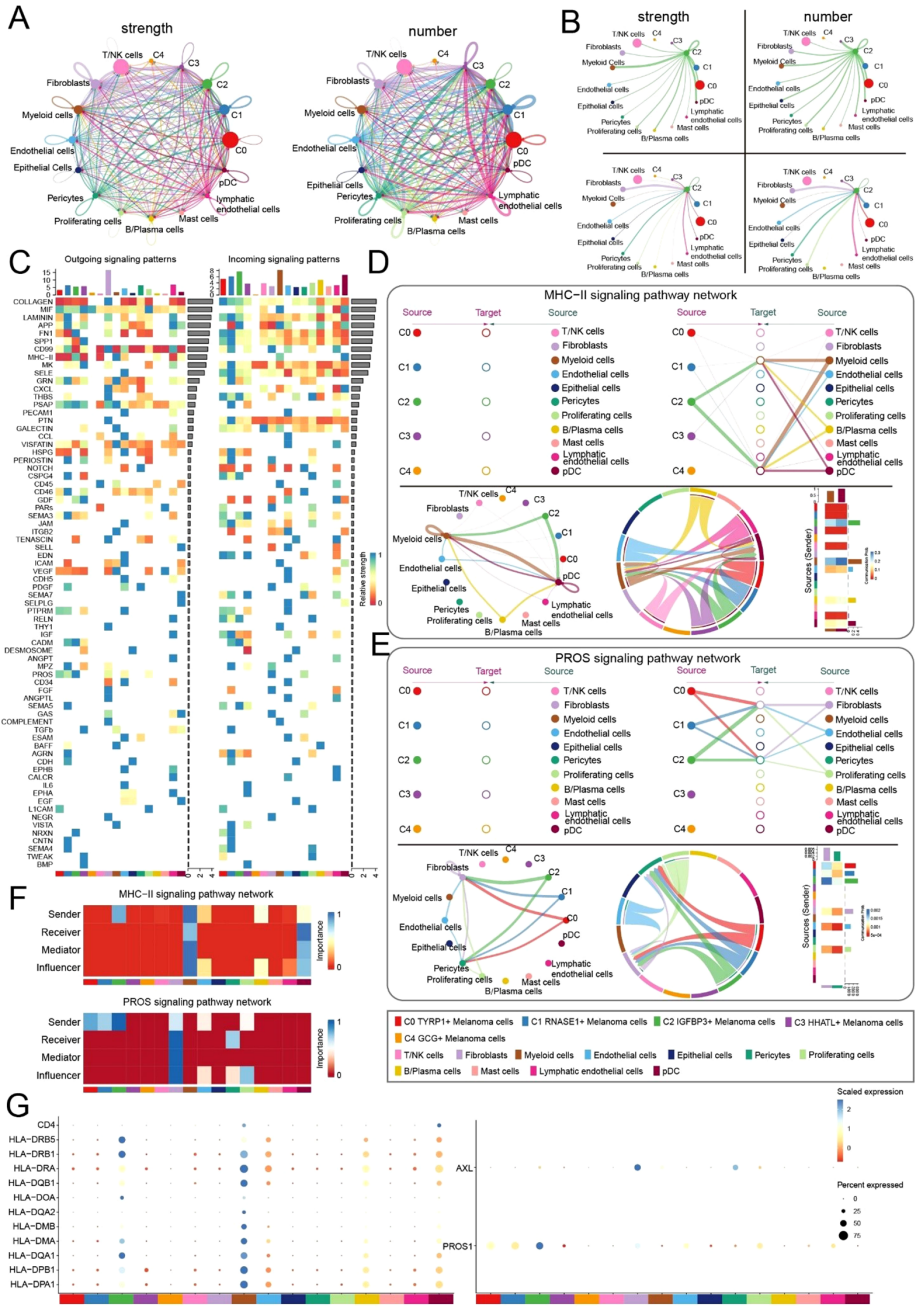
Microenvironment interaction network of *IGFBP3+* melanoma cell mediating MHC-II/PROS signaling pathways. **(A)** Circus plots showed the strength (left) and number (right) of interactions between melanoma cell subtypes and microenvironmental cells, with point size representing interaction quantity and line thickness reflecting communication probability.**(B)** Interaction analysis of C2 *IGFBP3+* melanoma cells with other cells: the upper left and upper right circle plots showed interaction strengths and numbers when acting as signal senders; the lower left and lower right circle plots showed interaction strengths and numbers when acting as signal receivers.**(C)** Heatmap of outgoing and incoming signaling patterns for melanoma cells.**(D)** Hierarchical diagram (upper), circle plot (lower left), chordal graph (lower middle) and heatmap (lower right) showed interactions between melanoma cell subtypes and other cell types in MHC-II signaling pathway.**(E)** Hierarchical diagram (upper), circle plot (lower left), chordal graph (lower middle) and heatmap (lower right) showed interactions between melanoma cell subtypes and other cell types in PROS signaling pathway.**(F)** Heatmap showed the cell communication centrality scores for MHC-II and PROS signaling pathways.**(G)** Bubble plots compared the ligand-receptor protein activity differences in the MHC-II (left) and PROS (right) pathways between melanoma cell subtypes and other cell types. .

Previous analyses have underscored the significant significance of the C2 subtype in melanoma; nonetheless, the complex mechanisms within the disease microenvironment require more examination. We conducted a number of analyses on cellular communication. Circle plots were utilized to depict the interaction intensity among the five melanoma subtypes and 11 supplementary cell types, thereby elucidating the fundamental attributes of the disease microenvironment (**Figure 5A**). We subsequently presented the interactions involving the C2 subtype, which functions as both the source and target of signaling, alongside other cell types, using a circle plot that illustrates the strength and quantity of these interactions (**Figure 5B**). A heatmap was utilized to depict the comparative intensity of outgoing and incoming signaling patterns among all cell types (**Figure 5C**). The results demonstrated that both MHC-II and PROS displayed elevated intensities in C2 outgoing signaling, however no intensities were detected in the incoming signaling patterns, indicating the need for additional exploration. We focused on these two signaling pathways for further investigation and depicted the interactions between melanoma cell subtypes and other cell types through various layouts. The C2 subtype interacted with myeloid cells and plasmacytoid dendritic cells inside the MHC-II pathway (**Figure 5D**). The C2 subtype within the PROS signaling pathway demonstrated interactions with fibroblasts and pericytes (**Figure 5E**). In the MHC-II and PROS signaling pathways, the C2 subtype mostly functioned as the Sender, whereas other cells predominantly served as the Receiver, Mediator, and Influencer (**Figure 5F**). We utilized bubble plots to depict the expression distribution of different ligand-receptor proteins throughout the two signaling pathways, therefore elucidating the distinct variations in ligand-receptor interactions (**Figure 5G**).

### 3.8 SCENIC regulatory patterns and critical TFs in melanoma cell subtypes

**Figure 6:**
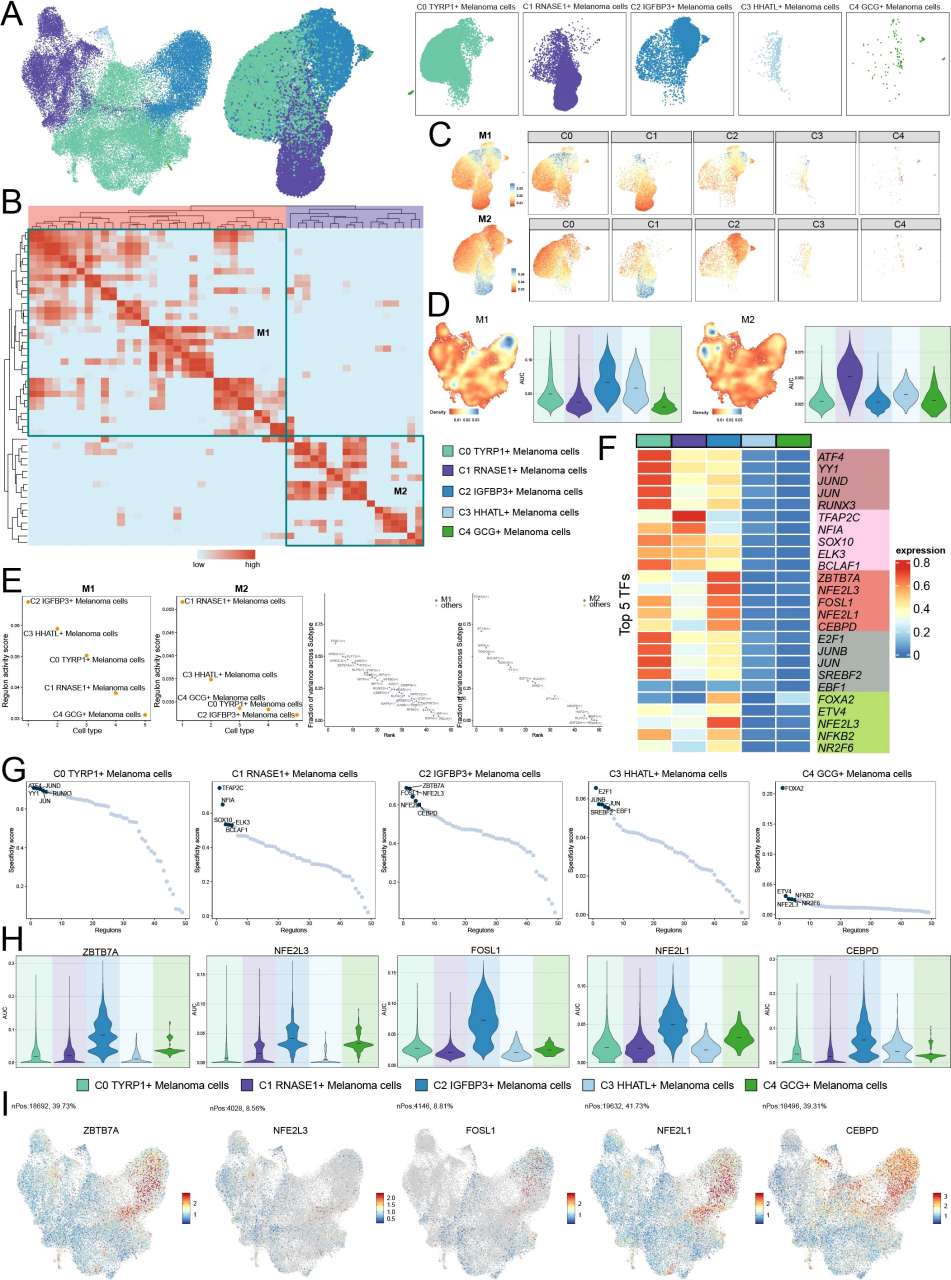
Transcriptional factor regulatory networks in melanoma cells. **(A)** Dimensionality reduction clustering of melanoma cells unsupervisedly (left) and based on all TF regulatory activities (right), visualized by faceted UMAP plots to show the distribution features of each subtype. **(B)** Heatmap based on SCENIC-identified transcription module similarity, using AUCell scores to identify two regulon modules (M1 and M2) in melanoma cells.**(C)** Faceted UMAP plots showed the distribution features of two regulatory modules across different melanoma cell subtypes.**(D)** UMAP plots illustrated the expressional density and violin plots further quantified the AUC across melanoma cell subtypes for each of the two modules.**(E)** Scatter plots on the left ranked subtypes based on regulon activity scores in each regulatory module, while plots on the right ranked regulons based on variance scores across the subtypes, with the key regulatory factors highlighted. **(F)** Heatmap showed the top five TFs in each melanoma cell subtype.**(G)** The scatter plots ranked the TFs of each melanoma cell subtype according to their regulon specificity score, with the top five ranked TFs highlighted.**(H)** Violin plots compared the activity differences of key TFs (ZBTB7A, NFE2L3, FOSL1, NFE2L1, CEBPD) in C2 *IGFBP3+* melanoma cell among the subtypes. **(I)** UMAP plots visualized the expressions of these TFs in C2 *IGFBP3+* melanoma cells.

We performed SCENIC analysis to further investigate the variability among different subtypes. Transcription factors were crucial in the regulation of gene expression and cellular activity. **Figure 6A** displayed a clustering analysis of distinct melanoma cell subtypes according to gene expression levels and transcription factor regulatory activities, whereas **Figure 6B** depicted the similarity of transcription modules found by SCENIC. The study identified two key patterns. We utilized UMAP analysis to evaluate the expression distribution of each subtype across different modules (**Figure 6C**). We subsequently displayed the densities and AUC ratings of these two modules across different subtypes. The results revealed that the C2 subtype exhibited increased expression activity in the M1 pattern (**Figure 6D**). The C2 subtype demonstrated the greatest regulon activity score within the M1 pattern. We evaluated the transcription factors across the subtypes according to the variance scores for each module (**Figure 6E**). We enhanced the primary transcription factors for different subtypes to examine their distinct regulatory pathways (**Figure 6F**). The C0 subtype primarily expressed ATF4, YY1, JUND, JUN, and RUNX3; the C1 subtype expressed TFAP2C, NFIA, SOX10, ELK3, and BCLAF1; the C2 subtype expressed ZBTB7A, NFE2L3, FOSL1, NFE2L1, and CEBPD; the C3 subtype expressed E2F1, JUNB, JUN, SREBF2, and EBF1; and the C4 subtype expressed FOXA2, ETV4, NFE2L3, NFKB2, and NR2F6. We subsequently displayed the five transcription factors with the best specificity ratings for each subtype (**Figure 6G**). We analyzed the differential expression distribution of the five predominant transcription factors in the C2 subtype using violin plots and UMAP (**Figures 6H, I**). The expression of FOSL1 in the C2 subtype was markedly increased compared to other cell subtypes. Furthermore, FOSL1 was the predominant transcription factor in the M1 pattern differentiating various subtypes. Nonetheless, the specific mechanism by which FOSL1 influences melanoma cells remains unclear. Consequently, it was imperative to conduct *in vitro* functional tests to evaluate the impact of FOSL1 on melanoma cells.

### 3.9 *In-vitro* experimental verification

To investigate the functional relevance of FOSL1 in melanoma cell proliferation and motility, we performed loss-of-function assays in A375 and MEWo cell lines with two distinct shRNAs targeting FOSL1 (sh-FOSL1–1 and sh-FOSL1-2). The knockdown efficiency was validated at both the mRNA and protein levels (**Supplementary Figure 1**).

**Figure 7:**
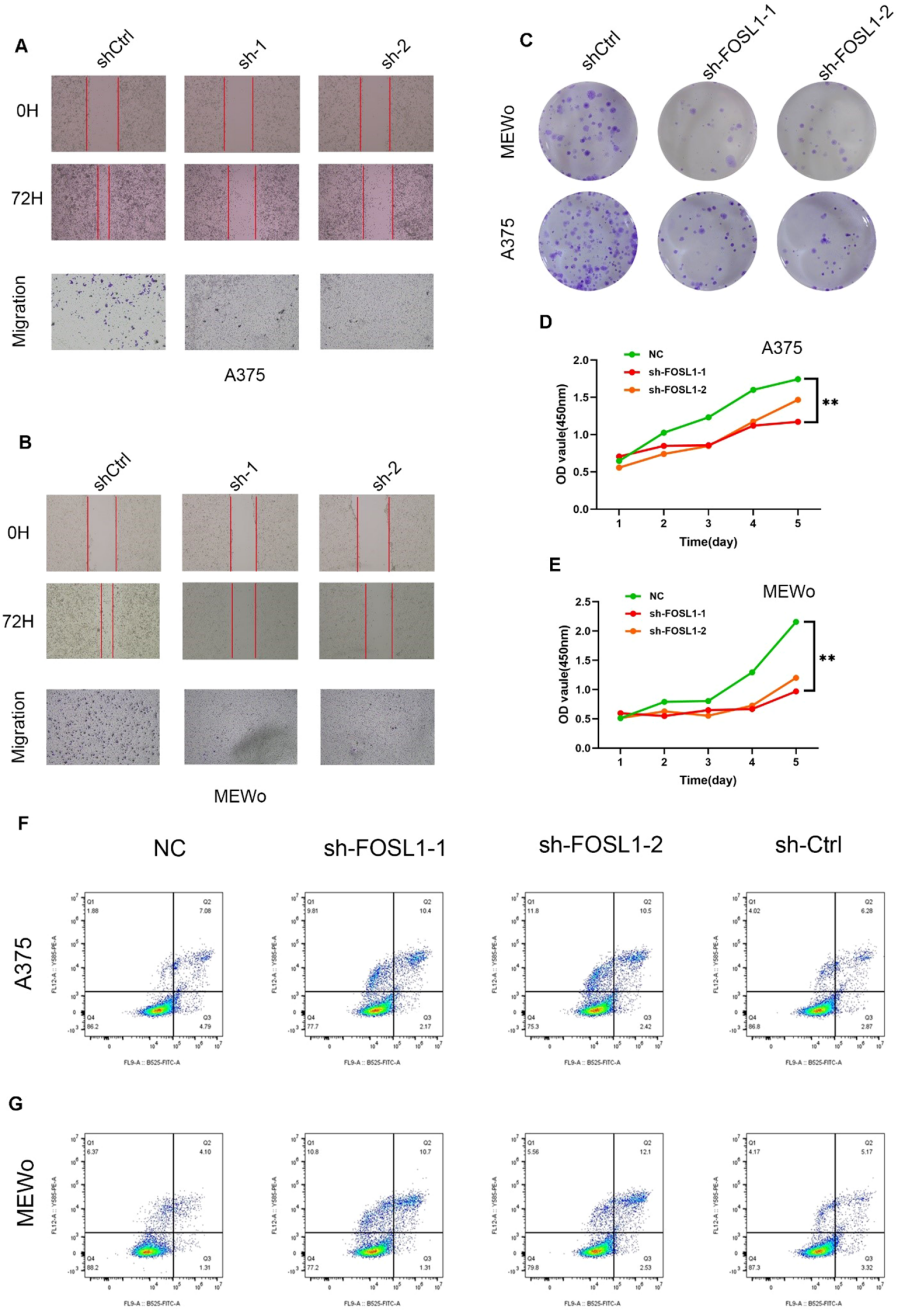
FOSL1 knockdown suppresses proliferation, migration, and promotes apoptosis in melanoma cells. **(A-B)** Representative images of wound healing assays at 0 h and 72 h, and transwell migration assays in A375 **(A)** and MEWo **(B)** cells following FOSL1 knockdown with two independent shRNAs (sh-1, sh-2). **(C)** Colony formation assay showing reduced clonogenic ability in A375 and MEWo cells after FOSL1 knockdown. **(D–E)** CCK-8 assays showing reduced proliferation in A375 **(D)** and MEWo **(E)** cells transduced with sh-FOSL1–1 or sh-FOSL1–2 over 5 days. P < 0.01 compared with NC group. **(F–G)** Flow cytometric analysis of apoptosis using Annexin V/PI staining in A375 **(F)** and MEWo **(G)** cells. Apoptotic populations increased upon FOSL1 knockdown. Data are presented as mean ± SEM. **, P < 0.01.

Wound healing and transwell experiments demonstrated that the silencing of FOSL1 greatly diminished the migrant capability of both A375 and MEWo cells in comparison to the shCtrl group (**Figures 7A, B**). Following FOSL1 knockout in both cell lines, clonogenic potential was constantly significantly decreased after colony formation experiments (**Figure 7C**).

The CCK- 8 test was used to assess cell viability over a duration of five days. **Figures 7D** and **Figure 7E** show that FOSL1 knockdown significantly inhibited cell proliferation in A375 and MEWo cells, indicating that FOSL1 has a growth-promoting role in melanoma.

Additionally, flow cytometry utilizing Annexin V/PI labeling demonstrated an elevation in apoptotic cell populations subsequent to FOSL1 silencing (**Figures 7F,G**), suggesting that FOSL1 may play a role in the survival of melanoma cells.

## 4 Discussion

Melanoma is considered the most deadly type of skin cancer due to its high tendency for metastasis and spread to other organs. The surgical excision of *in situ* tumors typically results in a curative effect. Managing metastatic illness poses significant hurdles (78–80). The advent of immunotherapy as a cancer treatment has transformed oncology and led to the implementation of novel, more efficacious treatment standards (81–83). This study identified a subtype enriched in advanced melanoma exhibiting a highly differentiated state and possesses immunoregulatory functions, which is predicted to simultaneously send dual signals via the MHC-II and PROS1 pathways, coordinating both the immune and neural microenvironments. The key transcription factor FOSL1 in this subtype drives the migration and survival phenotype and holds direct therapeutic potential. Five distinct tumor subtypes (C0–C4), each identified by distinct genetic markers and stage-specific enrichment patterns, were revealed in a single-cell study of 47, 047 melanoma cells. The C0 *TYRP1*+ subtype was generally enriched in early-stage tumors (Group I), confirming its probable role in melanogenesis and tolerance to oxidative stress during the early stages of carcinoma. In contrast, mid-stage tumors (Group II) showed increases in the C1 *RNASE1*+ and C4 *GCG*+ subtypes, which suggests that RNASE1 plays a role in the remodeling of extracellular matrix and dysregulated GCG signaling in the spread of disease. Advancement-stage cancers (Group III) showed a higher prevalence of theC2 *IGFBP3*+ and C3 *HHATL*+ subtypes, with IGFBP3 being linked to apoptosis resistance and HHATL-mediated hedgehog signaling, which might increase stemness. Cell-cycle analysis indicated the C4 subtype is very regenerative, with the highest CNV score and S score, suggesting an association with genetic instability and energetic DNA replication. In contrast, the C2 subtype exhibited reduced transcriptional activity (low nFeature/nCount RNA), possibly signifying a quiescent state. Additionally, the increase in C3 and C4 throughout the G2M/S phases highlights how crucially important they are in keeping proliferation in the illness’s later stages.

According to research using CytoTRACE2, subtype-specific diversity potentials were discovered in melanoma, indicating that the C2 subtype has the lowest differentiation potential, as demonstrated by a much decreased CytoTRACE2 score, which indicates a very differentiated state. C3 demonstrated the greatest differentiation potential, indicating a progenitor-like phenotype. This differentiation dichotomy was independent of tumor stage, indicating that differentiation hierarchies are predominantly determined by intrinsic subtype identity rather than disease progression. The stem cell properties of C3 might drive the evolution of advanced tumors. Gene profiling associated with stemness consistently demonstrated unique molecular characteristics: KLF4, a regulator of terminal differentiation, was elevated in C2, aligning with its differentiated state. Conversely, the pluripotency factors TWIST1, a master regulator of epithelial-mesenchymal transition, and MYC, a promoter of proliferation, were markedly elevated in C3, offering mechanistic understanding of its increased plasticity and undifferentiated characteristics. CytoTRACE2 showed the highest degree of C2 differentiation and late-stage enrichment, suggesting it might represent an endpoint state in tumor evolution. Although no direct evidence of transformation existed, C2 probably presented a stable state positively selected in advanced disease. Coexisting malignant pathways in advanced melanoma may elucidate the varied progression processes evident in stage III lesions.

Functional profiling identified the C2 subtype as an immunocompetent subtype of tumor cells in melanoma, distinctly enriched in immune regulatory mechanisms across all the ontological dimensions. C2 is specialized in cadherin binding and constructively regulates cell adhesion within biological processes, with its cellular components confined to the collagen-rich extracellular matrix and vacuolar membranes, which are essential for immunological synapse growth. C2 primarily governs T cell activation and leukocyte cell-cell adhesion at the molecular level, suggesting its potential function in directly influencing adaptive immunity. Convergent evidence from GSEA and network analysis has identified the MHC class II machinery as the central functional hub that facilitates peptide antigen assembly, MHC class II complex formation and lymphocyte-mediated immunity. This finding was further supported by the specific enhancement of B cell-centric pathways, which include the immunoglobulin-mediated reaction and B cell-mediated immunity, which suggests that C2 may be able to coordinate autoimmune inflammatory interactions. By believing works similar to those of antigen-presenting cells, C2 surpasses the traditional roles of tumor cells and establishes itself as a key component of the melanoma immune system. This may help to clarify the well-known potency of ICIs in recent years ‘ melanoma care. In people with advanced melanoma who received a combination of nivolumab and ipilimumab, a 10-year recurrence-free survival rate of 60% was found in a 2025 trial (84).

Our recognition of the immunoregulatory C2 *IGFBP3*+ subtype, predicted to act as a source of MHC-II and PROS pathways, raises questions about its potential role in the neuro-immune axis of melanoma. Given melanoma’s neural crest origin and established roles of nerves in shaping TME, it is plausible that this aggressive, immunomodulatory subtype interacts with or influences neural elements.

Especially interesting in the context of neuroimmunology is the strong MHC-II-mediated connection between C2 subtype and pDCs. pDCs can directly respond to neural signals by expressing receptors for various neurotransmitters and neuropeptides (85). Norepinephrine and other stress-induced neurotransmitters significantly impair pDC function, including IFN- and antigen-presenting abilities, potentially lowering anti-tumor immunity (86).Factors secreted by activated pDCs (e. g., IFNs, CXCL10) can influence neuronal activity and neuroinflammation conversely. The etiology of neuropathic problems has been linked to pDCs. Through cytokine release, they can infiltrate the sensory neurons and cause pain hypersensitivity (87, 88). The C2 subtype’s effective signaling to pDCs via MHC-II may affect TME’s device detection state, which may have an impact on patient quality of life and possibly immune function.

Critically, the PROS1-AXL signaling axis is not confined to cancer biology but plays important roles within nervous system and neuroinflammation. The AXL receptor tyrosine kinase is expressed on neurons, microglia, Schwann cells and astrocytes (89). In physiological contexts, PROS1/Gas6 binding to AXL on microglia exerts potent anti-inflammatory effects suppressing microglial activation and promoting efferocytosis (90). However, tumor-driven activation of this pathway on stromal cells likely exploits these immunosuppressive and pro-survival mechanisms to foster an immune-tolerant niche within melanoma TME. Furthermore, AXL signaling in Schwann cells and neurons is implicated in neuropathic pain, which is a common and debilitating comorbidity in advanced cancer patients (91). In conjunction with active PROS1 signaling, C2 might contribute to cancer-related pain, a major determinant of patients’ quality of life. The potential impact of C2-derived PROS1 signaling on peritumoral nerves or pain perception and the expression mechanisms of pain-related genes such as NGF and BDNF warrant investigation.

Based on our ligand-receptor analysis, C2 did not significantly express neuronal receptors (such as ADRB2). Unlike known direct tumor innervation, the C2 subtype activates pDCs (responsive to neural signals) via the MHC-II pathway and regulates stromal cells (involved in neurorepair) via the PROS pathway. We suggested that the C2 subtype might have an indirect effect on neuronal components. Its PROS1 signaling to fibroblasts/pericytes may modify the matrix environment that supports nerve fibers, and its interaction with pDCs through MHC-II likely responds to neurological signals. Functional interactions are still a prediction that needs to be confirmed in the future. Furthermore, our findings added critical complexity to the dichotomous view of classifying the melanoma immune microenvironment as either “immune-infiltrated” or “immune-desert” (92). The C2 subtype might actively participate in shaping the immune environment. By potentially engaging and modulating T cells and antigen-presenting cells, it leads to a dysfunctional, immunosuppressive form of “immune-infiltrated” microenvironment, which probably underlies the mixed response of patients to immunotherapy. This perspective is supported by recent studies highlighting the prognostic importance of MHC-II expression in tumors themselves and its complex role in immune evasion (93, 94).

The transcriptional regulatory profiles clarify the primary factors influencing melanoma subtype differentiation, with the C2 subtype defining its immunoreactive identity via a unique M1 regulatory module marked by elevated regulon activity scores. This module is primarily regulated by TFs including ZBTB7A, NFE2L3, and FOSL1, with FOSL1 identified as a distinctive regulatory factor for C2, with markedly elevated expression levels relative to other subtypes. Functional experiments have established that FOSL1 is an essential determinant in promoting the fundamental malignant characteristics of melanoma. In A375 and MEWo cell lines, the knockdown of FOSL1 led to a significant decline in migratory ability (demonstrated by wound healing and transwell assays), a reduction exceeding 60% in clonogenic formation, a flattening of CCK-8 proliferation curves, and a 2.8-fold increase in apoptosis as indicated by Annexin V^+^ cells. The phenotypic changes were strongly aligned with predictions from single-cell regulatory network analysis.

FOSL1, a significant part of the AP-1 transcription factor family, has attracted a lot of attention because of its role in the progression of melanoma. According to research, FOSL1 expression is significantly higher in melanoma patient samples than nevi (95), and this higher level of expression is related to shorter patient survival (96). FOSL1 expression levels are generally elevated in patients with metastatic melanoma (97). Aberrant expression of FOSL1 has been recorded in melanoma cell lines, suggesting its possible involvement in melanoma development (96). Through its specific protein HMGA1 (95), FOSL1 facilitates melanoma cell proliferation and migration by controlling cytoskeletal rearrangement and extracellular matrix interactions at the molecular level. Furthermore, FOSL1 can affect Cyclin E or regulate apoptosis-related proteins to promote melanoma cell proliferation (98, 99). Moreover, FOSL1 may augment the migratory and invasive characteristics of melanoma cells by activating pertinent signaling pathways including the PI3K-Akt pathway (99). Prior research has shown that inhibiting FOSL1 expression via gene editing methods or small molecule inhibitors in cellular and animal models, markedly hinders the proliferation, migration and invasive potential of melanoma cells, while also diminishing tumor growth and metastasis (100). Moreover, FOSL1 is implicated in neuroinflammation (101, 102). Previous studies have focused on the metastasis-promoting mechanism of FOSL1. This study revealed its specific expression in immunoregulatory subtypes. Its potential neuro-immunological effects and regulatory roles in immunoregulatory pathways require further validation in future studies. Investigating whether FOSL1 knockdown alters the expression profile of nerves or neuroimmune signaling molecules could reveal novel neuro-immunomodulatory functions of this oncogenic TF in melanoma.

The development of single-cell sequencing technology has transformed biological research by facilitating the thorough investigation of individual cells, offering important insights into cell heterogeneity and the complex molecular pathways that underlie illnesses like melanoma (103). With the aid of specialized treatment regimens, this technology has improved our understanding of the tumor microenvironment and led to the identification of specific subtypes that contribute to the development of disease or therapeutic resistance (104). Single-cell analysis, in addition to offering a more in-depth view of tumor heterogeneity, has improved our understanding of melanoma heterogeneity. The C2 *IGFBP3*+ tumor cell subtype discovery raises important questions for upcoming study. The inquiry into this subtype resulted in formulation of tailored approaches for early screening and therapy, encompassing the identification of prospective biomarkers to augment detection and therapeutic targets to improve treatment success. Although our study establishes strong links between the C2 type and FOSL1 in the development and immunomodulation of melanoma, there are limitations to our research. The limited sample size may have hampered the representativeness of the effects because the focus was on single-cell information from a particular type of melanoma people. Moreover, the current scRNA-seq analysis concentrated on studying immune-stromal interactions and biological diversity without specifically capturing glial or neuronal cell populations or analyzing neural activity markers. Potential studies incorporating spatial transcriptomics or multi- omics approaches that include cerebral markers are necessary to instantly map the geographical relationship between the C2 *IGFBP3+* subtype, nerves and immune cells within the melanoma TME. Also, functional experiments exploring how neurotransmitters, neuropeptides or ablation of specific nerves affects the C2 subtype’s phenotype, FOSL1 expression and its immunomodulatory capacity are warranted to establish direct links within the neuro-immune axis. Another promising direction forward involves integrating neuroimmunology perspectives, such as studying the effects of chronic stress models (known to modulate neurotransmitters and immunity) on C2 subtype dynamics and FOSL1 expression.

This research examined the heterogeneity of tumor cells in melanoma at the single-cell level, highlighting the significant involvement of FOSL1. Furthermore, we identified the signaling pathway related with antigen presentation in the immune system. More research is needed to understand how FOSL1 and the immunological environment interact. Our discoveries have made improvement in comprehending the biology of melanoma and opened up new avenues for the treatment and prevention of illness. These studies should be expanded upon in future research to improve understanding and treatment of melanoma. The use of scRNA-seq in fundamental and translational research has advanced personalized therapy by finding new therapeutic targets for novel drug development, revealing biomarkers for monitoring treatment efficacy and informing therapeutic decision-making.

## 5 Conclusion

In conclusion, we identified a distinct, predicted-immunoregulatory *IGFBP3+* melanoma cell subtype and established FOSL1 as a key oncogenic driver within this subtype. Targeting FOSL1 or cytotherapy targeting C2-associated pathway markers hold significant therapeutic promise. Importantly, the signaling networks employed by the C2 *IGFBP3+* subtype, particularly the PROS and MHC-II pathway, suggest a potential, unexplored role for this aggressive subtype in mediating neuro-immune crosstalk within the melanoma microenvironment. Elucidating these neuro-immunological interactions represents a critical next step in fully understanding melanoma progression and resistance, paving the way for novel combinatorial strategies that co-target the tumor, immune, and nervous systems.

## Data Availability

The original contributions presented in the study are included in the article/supplementary material. Further inquiries can be directed to the corresponding author/s.
