# Supplementary material for: Single-cell transcriptomics identifies FOSL1-regulated IGFBP3+ melanoma subtype as a neuro-immunoregulatory signaling hub facilitating tumor progression
Source: Front Immunol. 2025 Nov 26;16:1662869. doi: 10.3389/fimmu.2025.1662869 (PMC12689971; doi:10.3389/fimmu.2025.1662869)
Supplement: Supplementary file 1 [file SupplementaryFile1.docx]

Supplementary Material

# Supplementary Figures


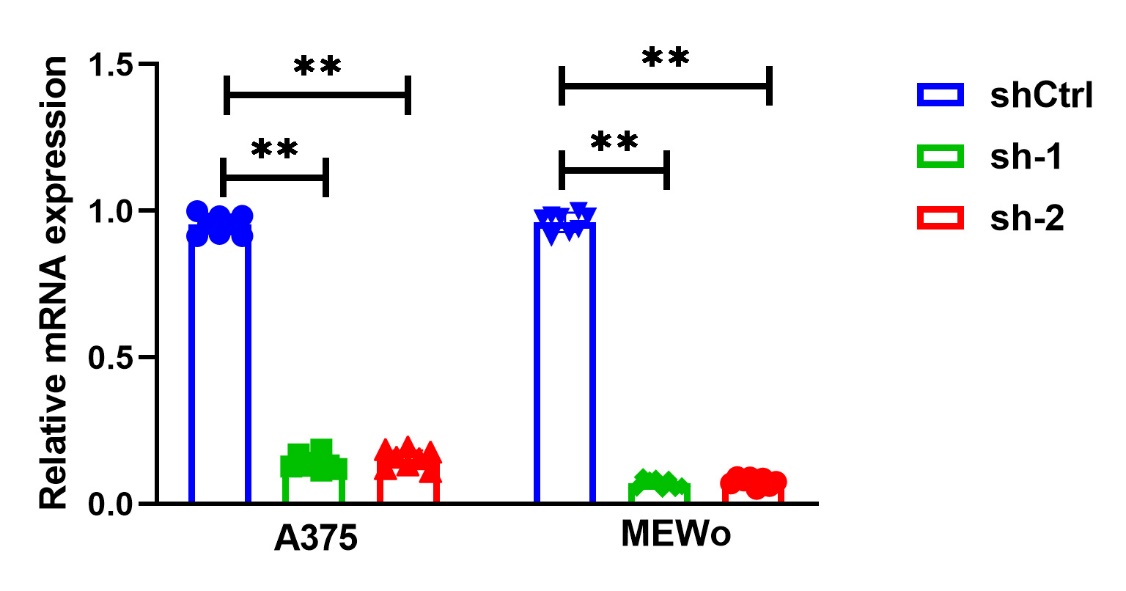


**Supplementary Figure 1.** Relative expression of FOSL1 was significantly lower in sh-1 and sh-2 compared to shCtrl after knockdown. **: P < 0.01.

# Supplementary Tables

**Supplementary Table 1. qRT-PCR primer sequences of FOSL1 and GAPDH**

|  | **Sequence** |
| --- | --- |
| **FOSL1 forward** | 5′-CAGCTGACAGCCTTTGACTT-3′ |
| **FOSL1 reverse** | 5′-TGCTTGATGATGTGCTGAGG-3′ |
| **GAPDH forward** | 5′-GGAGCGAGATCCCTCCAAAAT-3′ |
| **GAPDH reverse** | 5′-GGCTGTTGTCATACTTCTCATGG-3′ |
